# Progesterone Attenuates Allodynia of Inflamed Temporomandibular Joint through Modulating Voltage-Gated Sodium Channel 1.7 in Trigeminal Ganglion

**DOI:** 10.1155/2020/6582586

**Published:** 2020-07-25

**Authors:** Rui-Yun Bi, Xiao-Yu Zhang, Peng Zhang, Yun Ding, Ye-Hua Gan

**Affiliations:** ^1^Third Clinical Division, Peking University School and Hospital of Stomatology & National Clinical Research Center for Oral Diseases & National Engineering Laboratory for Digital and Material Technology of Stomatology & Beijing Key Laboratory of Digital Stomatology, 10 Huayuan Lu, Haidian District, Beijing 100088, China; ^2^Central Laboratory, Peking University School and Hospital of Stomatology & National Clinical Research Center for Oral Diseases & National Engineering Laboratory for Digital and Material Technology of Stomatology & Beijing Key Laboratory of Digital Stomatology, 22 Zhongguancun South Avenue, Haidian District, Beijing 100081, China; ^3^Center for TMD & Orofacial Pain, Peking University School and Hospital of Stomatology & National Clinical Research Center for Oral Diseases & National Engineering Laboratory for Digital and Material Technology of Stomatology & Beijing Key Laboratory of Digital Stomatology, 22 Zhongguancun South Avenue, Haidian District, Beijing 100081, China

## Abstract

**Background:**

Women with temporomandibular disorders (TMDs) experience some amelioration of pain during pregnancy. Progesterone increases dramatically and steadily during pregnancy. Sodium channel 1.7 (Nav1.7) plays a prominent role in pain perceptions, as evidenced by deletion of Nav1.7 alone leading to a complete loss of pain. In a previous study, we showed that Nav1.7 in trigeminal ganglion (TG) is involved in allodynia of inflamed temporomandibular joint (TMJ). Whether progesterone modulates allodynia of inflamed TMJ through Nav1.7 in TG remains to be investigated.

**Methods:**

The effects of progesterone on sodium currents of freshly isolated TG neurons were examined using whole-cell recording. Female rats were ovariectomized and treated with increasing doses of progesterone for 10 days. Complete Freund's adjuvant was administered intra-articularly to induce TMJ inflammation. TMJ nociceptive responses were evaluated by head withdrawal thresholds. Real-time PCR and Western blotting were used to examine Nav1.7 mRNA and protein expression in TG. Immunohistofluorescence was used to examine the colocalization of progesterone receptors (PR*α*/*β*) and Nav1.7 in TG.

**Results:**

Whole-cell recording showed that progesterone could attenuate sodium currents. Moreover, progesterone dose-dependently downregulated Nav1.7 mRNA expression and reduced the sensitivity of TMJ nociception in ovariectomized rats. Furthermore, treatment with progesterone attenuated allodynia of inflamed TMJ in a dose-dependent manner and repressed inflammation-induced Nav1.7 mRNA and protein expression in ovariectomized rats. The progesterone receptor antagonist, RU-486, partially reversed the effect of progesterone on allodynia of inflamed TMJ and TMJ inflammation-induced Nav1.7 mRNA and protein expression.

**Conclusion:**

Progesterone, by modulating trigeminal ganglionic Nav1.7, may represent a promising agent to prevent allodynia of inflamed TMJ.

## 1. Introduction

Temporomandibular disorders (TMDs) have the highest prevalence in women of reproductive age, implying that sex hormones may be involved in TMD pain processing [1, 2]. Previously, we have reported that estrogen could aggravate temporomandibular joint (TMJ) inflammation and pain [3]. However, it is difficult to explain why women with TMDs experience some amelioration of pain during pregnancy [4, 5], even though estrogen levels are prominently increased throughout pregnancy. A case-control study found the prevalence of TMDs in the nonpregnant group was 45.5% and only 15.2% in the pregnant group. The considerably lower prevalence of these disorders in pregnant women suggests that pregnancy protects against TMD signs and symptoms [4]. Similar to estrogen, progesterone also increases dramatically and steadily during pregnancy. Moreover, women without any TMDs were found to have a higher progesterone level (11.6 ± 10.4 ng/ml) compared to women with TMDs (8.4 ± 6.8 ng/ML) [6]. Thus, the higher progesterone level in women without TMDs suggests progesterone may improve TMD symptoms.

The effects of progesterone on nociception are complex, as with estrogen [7, 8]. Neurons and glial cells of the brain, the thoracic ganglion, the protocerebrum, the subesophageal ganglion, the leg ganglion, and spinal cord express various progesterone receptors (PR) [9], including the classical PRs, PR*α,* and *β*, making these cells sensitive targets of this steroid. Numerous studies have reported that progesterone either does not affect nociception or decreases nociception; the different effects of progesterone on nociception may be due to the testing site and the pattern of progesterone administration [7, 10–13]. Moreover, progesterone was demonstrated to decrease neuron excitability through direct inhibition of voltage-gated potassium channels, calcium channels, and sodium channels [14]. These studies suggest that progesterone may play a role in pain perception. Therefore, whether and how progesterone contributes to TMJ inflammation and pain remains unclear.

The voltage-gated sodium channel 1.7 (Nav1.7) plays a prominent role in pain perception. Three human pain syndromes are caused by the mutations of this gene: congenital inability to experience pain [15], primary erythromelalgia [16], and paroxysmal extreme pain disorder [17]. Nav1.7 is highly expressed in the sympathetic ganglia, dorsal root ganglia (DRG), TG, and other pain sensing nerves [18], and it can amplify weak stimuli in these neurons and act as the threshold channel for firing action potentials [19]. Nav1.7 also plays an important role in inflammatory pain. Both herpes vector-mediated knockdown of Nav1.7 in primary afferents in mice and nociceptor-specific knockout of Nav1.7 in mice attenuated inflammation-induced allodynia [20, 21]. Furthermore, our previous study have used fluorescent retrograde neuronal tracer DiI to label the TG neurons innervating the TMJ and showed that trigeminal ganglionic Nav1.7 was specifically upregulated in the neurons innervating TMJ after the induction of TMJ inflammation [22].

Therefore, we hypothesized that trigeminal ganglionic Nav1.7 might be involved in progesterone-mediated attenuation of pain caused by TMJ inflammation. Thus, ovariectomized rats were injected progesterone at doses of 0 *μ*g, 350 *μ*g, or 700 *μ*g to simulate the physiologic progesterone swings of estrous cycle in normal female rats, and then, we explored whether progesterone could attenuate TMJ pain and whether progesterone could modulate Nav1.7 expression in TG to attenuate TMJ allodynia.

## 2. Methods

### 2.1. Animals

In this study, adult female Sprague–Dawley (SD) rats (180–200 g) were used. The experimental protocols were approved by the Peking University Biomedical Ethics Committee Experimental Animal Welfare Ethics Branch (No. LA2008-004, Beijing, China). The rat room was kept clean, quiet, and uncluttered. The rats were housed under 22°C ± 1°C on a 12 : 12 h light/dark cycle and were provided ad libitum access to food and water. Animals were monitored at least once daily, including weekends and holidays, and animals were monitored at least twice daily after recovering from anesthesia, or surgery for ovariectomy, or induction of TMJ inflammation, or replacement of progesterone (P4), or progesterone receptor antagonist administration. In this study, ninety-six rats were sacrificed, and two of them died under anesthesia based on the same anesthesia treatment (1% sodium pentobarbital, 40 mg/kg, i.p.) and same care. However, it was possible that some of them may have died of individual sensitive responding to the anesthesia. No animals became severely ill. All efforts were made to minimize animal suffering. At the end of each experimental test, the rats were euthanized by an anesthesia overdose with 1% pentobarbital sodium (100 mg/kg body weight).

### 2.2. Drugs

The drugs used in this study—Complete Adjuvant of Freund (CFA, temporomandibular inflammation inducer), P4, and mifepristone (RU486, the progesterone receptor antagonist)—were purchased from Sigma-Aldrich.

### 2.3. Animal Group Dividing and Treatments

To examine the effects of P4 on TMJ pain, female rats (180 g–200 g) were divided into 4 groups (*n* = 7), as follows: control group (sham-ovariectomized rats treated with vehicle for 10 days), 0 *μ*g P4 group (ovariectomized rats treated with vehicle for 10 days), 350 *μ*g P4 group (ovariectomized rats treated with 350 *μ*g P4 for 10 days), and 700 *μ*g P4 group (ovariectomized rats treated with 700 *μ*g P4 for 10 days).

To test the effects of both P4 and inflammation on TMJ pain, female rats (180 g to 200 g) were divided into 5 groups (*n* = 7) as follows: control group (sham-ovariectomized rats treated with vehicle for 10 days), sham + CFA group (sham-ovariectomized rats treated with vehicle for 10 days and injected with 50 *μ*l complete Freund adjuvant (CFA) into the bilateral TMJs to induce TMJ inflammation), 0 *μ*g P4 + CFA group (ovariectomized rats treated with vehicle and injected with 50 *μ*l CFA into the bilateral TMJs), 350 *μ*g P4 + CFA group (ovariectomized rats treated with 350 *μ*g P4 and injected with 50 *μ*l CFA into the bilateral TMJs), and 700 *μ*g P4 + CFA group (ovariectomized rats treated with 700 *μ*g P4 and injected with 50 *μ*l CFA into the bilateral TMJs). TMJ inflammation was induced on the tenth day of P4 treatment, and the control rats were injected with vehicle (50 *μ*l saline).

To examine the effects of progesterone receptors on TMJ pain, female rats (180 g to 200 g) were divided into 4 groups (*n* = 7), as follows: a control group (sham-ovariectomized rats treated with vehicle for 10 days), an inflammation group (sham-ovariectomized rats treated with vehicle for 10 days), a 700 *μ*g P4 group (ovariectomized rats treated with 700 *μ*g P4), and a 700 *μ*g P4 + RU486 group (ovariectomized rats treated with 700 *μ*g P4 and RU486). The last three groups were injected with 50 *μ*l CFA into the bilateral TMJs to induce TMJ inflammation, while the control group was injected with vehicle (50 *μ*l saline).

### 2.4. Cell Preparation and Patch Clamp Recording

Freshly isolated TG neurons were obtained from 180 to 200 g female SD rats (*n* = 3), as previously described [23] with slight modification. Briefly, TGs were isolated and collected in 4°C L15 medium and were cut into pieces to be digested later with collagenase IA (Sigma Chemicals, St. Louis, MO; 1 mg/ml) and trypsin I (Sigma Chemicals, St. Louis, MO; 0.3 mg/ml) at 37°C for 40 min. Individual cells were dissociated by triturating through pipettes and then were plated and incubated onto poly-L-lysine pretreated dishes containing F12 medium. Since Nav1.7 was mainly located in the small neurons and medium neurons as shown our previous study [3, 22], TG neurons (15–25 *µ*m in diameter) were used 1–8 h after preparation or cultured for 24 h before use.

Whole-cell recording was performed using an EPC/10 amplifier (HEKA Elektronik, Lambrecht/Pfalz, Germany) and Pulse Software. Patch pipettes with 3-4 M were used during the voltage clamp, and cells were held at −80 mV. Data were analyzed with Igor software (Wavemetrics, Lake Oswego, OR). Significance changes were tested using the two-tailed unpaired Student's *t*-test (^*∗*^*P* < 0.05). For sodium current recording, the extracellular solution contained (in mM) the following: 5 KCl, 150 NaCl, 0.2 CdCl_2_, 2.5 CaCl_2_, 10 HEPES, 10 glucose, and 20 TEA-Cl (pH 7.4 adjusted with NaOH). 20 mM tetraethyl ammonium chloride (TEA-Cl) and 0.2 mM CdCl_2_ were puffed extracellularly to block endogenous voltage-gated potassium currents and calcium currents; the intracellular solution contained (in mM) the following: 107 CsF, 1 CaCl_2_, 10 NaCl, 2 MgCl_2_, 10 TEA-Cl, 10 HEPES, 10 EGTA, and 4 NaATP (pH 7.2 with CsOH). Progesterone (P4) was stocked at 50 mM in ethanol and diluted to 50 *µ*M with recording extracellular solution for use. To study steady-state activation, TG cells were activated to various test potentials for 50 ms from a holding potential of −100 mV. Peak sodium conductance (*G*) was normalized to the maximal peak conductance (*G*_max_) and then fit to the Boltzmann function of the form *G*/*G*_max_ = (1 + exp ((*V*_m_ − *V*_½_) *k*)) − 1 (*V*_m_, membrane potential; *V*_½_, half-activated voltage; *k*, slope factor). To measure steady-state inactivation, cells were held at a prepulse potential from −120 mV to 30 mV for 500 ms and then subjected to a test pulse of −10 mV for 50 ms. Normalized peak currents were fit by the Boltzmann function *I*/*I*_max_ = (1 + exp ((*V*_m_ − *V*_½_) *k*)) − 1, and the *I*_max_ was the peak current activated at −10 mV after the prepulse of −120 mV.

### 2.5. Induction of TMJ Inflammation

CFA (oil/saline at ratio of 1 : 1, 0.025 mg *Mycobacterium tuberculosis*, Sigma) was injected into the bilateral TMJs to induce TMJ inflammation for 24 h as described in detail previously [22, 24].

### 2.6. Measurement of Head Withdrawal Threshold

Behavioral testing was conducted on a blind basis, in which the investigators were blinded to the rat treatment. The head withdrawal threshold, which is negatively associated with the mechanical sensitivity of the orofacial region [25], was conducted as reported previously [22, 24, 26, 27], and the results were calculated as mean ± standard deviation (SD) based on 5 measurements per joint and 4 rats per group. Briefly, the rats were habituated to rear on their hind paws and recline against the experimenter's working glove. The rats can move freely but were kept motionless during the test session. At 2 h before rats were sacrificed, the tip of the filament of an electronic von Frey anesthesiometer (IITC Life Science, Woodland Hills, CA, USA) was placed on the skin above the TMJ with progressive, increasing forces to the TMJ region until the head was withdrawn; the applied force was automatically recorded.

### 2.7. P4 Administration

After being anesthetized with 1% pentobarbital sodium (50 mg/kg body weight) administered intraperitoneally, rats were bilaterally ovariectomized or sham-operated (control and sham-ovariectomized groups) and allowed to recover for 1 week. The P4 was dissolved in ethanol and diluted to 10% in saline at a volume of 100 *μ*l immediately before administration. The groups of ovariectomized rats were treated with P4, administered by subcutaneous abdominal injection daily in the morning, at doses of 350 *μ*g or 700 *μ*g P4 per rat, depending on group, for 10 days. The same amount of vehicle was injected in the control group, the sham groups, and the group receiving 0 *μ*g P4. In the experimental groups, rats in the sham-ovariectomized and ovariectomized groups received 50 *μ*l injections of Freund's complete adjuvant (CFA; Sigma) (1 : 1 oil : saline emulsion) into each of the TMJs to induce bilateral TMJ inflammation for 24 hours on the tenth day of P4 treatment. Rats in the control group received 50 *μ*l injections of saline into each of the TMJs.

### 2.8. P4 Determination

**Figure 1:**
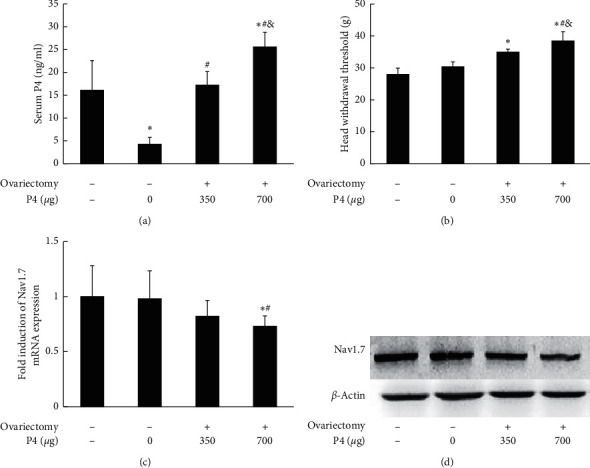
Progesterone dose dependently attenuated sensitivity of TMJ nociception and downregulated Nav1.7 expression in TG. (a) Confirmation of the effectiveness of ovariectomized and P4 replacement in rats. Serum levels of P4 in the ovariectomized groups treated with 0 *μ*g, 350 *μ*g, or 700 *μ*g P4 increased dose dependently. (b) Head withdrawal threshold. The lower the head withdrawal thresholds, the more sensitive the TMJ nociception will be. The head withdrawal threshold was increased by P4 application, suggesting that the sensitivity of TMJ nociception was decreased. (c) Real-time PCR analysis for Nav1.7 expression in TG. P4 increased the mRNA expression of Nav1.7 in TG. (d) Representative immunoblotting for Nav1.7 expression in TG after estrogen replacement. *β*-Actin served as an internal control for equal loading. ^*∗*^*P* < 0.05 versus the control group; ^#^*P* < 0.05 versus the 0 *μ*g group; ^&^*P* < 0.05 versus the 350 *μ*g P4 group. Data are expressed as mean ± SD.

**Figure 2:**
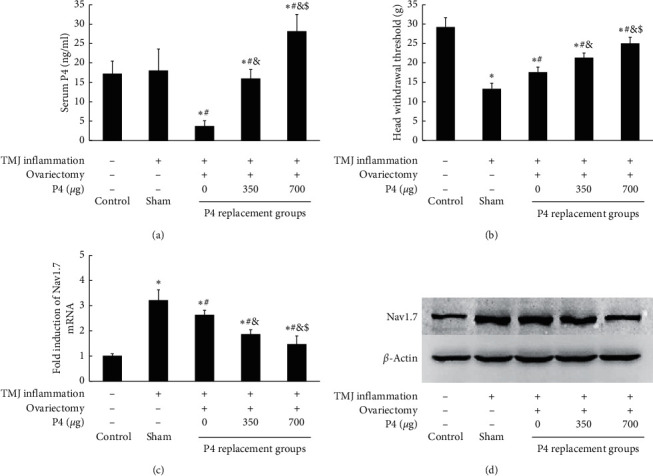
Progesterone attenuated TMJ inflammation-induced hyperalgesia and Nav1.7 expression. (a) Serum level of P4. Serum levels of P4 in the ovariectomized groups administered 0 *μ*g, 350 *μ*g, or 700 *μ*g P4 increased dose dependently. (b) Head withdrawal threshold. After induction of TMJ inflammation, the head withdrawal threshold was significantly decreased in sham rats and also dose-dependently increased in the ovariectomized groups. (c) Real-time PCR analysis for Nav1.7 expression in TG. The Nav1.7 mRNA in TG was increased by TMJ inflammation and attenuated dose-dependently by P4 in ovariectomized rats. (d) Representative immunoblotting for Nav1.7 expression in TG. Nav1.7 protein expression corresponded to mRNA expression pattern. *β*-Actin served as an internal control for equal loading. ^*∗*^*P* < 0.05 versus the control group; ^#^*P* < 0.05 versus the sham group; ^&^*P* < 0.05 versus the 0 *μ*g group; ^&^*P* versus the 350 *μ*g group. Data are expressed as mean ± SD.

Blood was obtained from those ovariectomized rats with different P4 administration or in the experimental groups 24 hours after induction of TMJ inflammation. The effectiveness of ovariectomy and P4 replacement was confirmed by the measurement of serum P4 as shown in Figures 1(a) and 2(a), and the serum levels of P4 were measured by radioimmunoassay using a Beckman Coulter Access immunoassay system.

### 2.9. Application of Progesterone Receptor Antagonist

The progesterone receptor specific antagonist, RU486, was dissolved in ethanol and diluted to 10% in saline at a volume of 100 *μ*l immediately before administration. Female rats were divided into 4 groups (control, inflammation, 700 *μ*g P4 group, and 700 *μ*g P4 + RU486 group); the control, inflammation, and 700 *μ*g P4 groups were intraperitoneally injected with vehicle, and the 700 *μ*g P4 + RU486 group was intraperitoneally injected with RU-486 (5 mg/Rat) twice in 24 hours, before and immediately after CFA injection. The application and dose of RU-486 were modified from those used in previous studies [28, 29]. The head withdrawal threshold was measured 2 hours before (baseline) and 22 hours after CFA injection. The TG was dissected 24 hours after CFA injection.

### 2.10. Real-Time Quantitative PCR and Western Blot Analysis

The mRNA expression of Nav1.7 in TG was detected as described previously [3, 22]. Briefly, total RNA was extracted with TRIzol (Invitrogen, Carlsbad, CA); reverse transcription was conducted with the iScript cDNA synthesis kit (Bio-Rad, Hercules, CA), and real-time PCR was performed with Power SYBRGreen PCR Master Mix (Applied Biosystems, Foster City, CA) using the 7500 real-time PCR system (Applied Biosystems). The efficiency of primers for rat *β*-actin and Nav1.7 was confirmed previously [22].

The protein expression of Nav1.7 in TG was assessed via Western blotting following a detailed method previously described [22]. The primary antibodies were anti-Nav1.7 antibody (1 : 500, AB5390, Millipore, USA) and anti-*β*-actin antibody (1 : 1000, sc-1616-R, Santa Cruz, USA).

### 2.11. Immunohistofluorescence

Intact female rats were anesthetized with an overdose of pentobarbital sodium and euthanized by transcardiac perfusion (250 ml body temperature, 0.1 M PBS, and pH 7.4, followed by 200 ml–300 ml ice-cold 4% paraformaldehyde in 0.1 M PBS and pH 7.4). After perfusion, TGs were postfixed in 4% paraformaldehyde for 4 h, incubated in 30% sucrose solution (in 0.1 M PBS) overnight at 4°C, frozen to −20°C, and sectioned 5 *μ*m thick on a cryostat. The sections were then mounted on poly-L-lysine-coated slides, blocked with 10% goat serum for 30 min at room temperature, and incubated with primary antibody overnight at 4°C. Rabbit polyclonal anti-Nav1.7 antibody (1 : 500, AB5390, Millipore, USA) and mouse monoclonal anti-PR*α*/*β* antibody (1 : 500, SC-810, Santa Cruz Biotechnology, USA) were used correspondingly as primary antibodies to evaluate the colocalization of Nav1.7 and PR*α*/*β*. After extensive washing with PBS, the sections were incubated with tetramethyl rhodamine isothiocyanate-conjugated anti-rabbit or fluorescein isothiocyanate-conjugated anti-mouse secondary antibody (1 : 200; Jackson ImmunoResearch, Santa Cruz, CA, USA) for 30 min at room temperature and then washed with PBS. The sections were counterstained with 4′,6-diamidino-2-phenylindole for 1 min (5 mg/ml), washed three times with PBS, and covered with fluorescence mounting medium. Confocal microscopic images were acquired using a Zeiss laser scanning microscope (LSM 510, Carl Zeiss, Jena, Germany), and the images were processed using the LSM 5 Release 4.2 software. Our previous study has used fluorescent retrograde neuronal tracer DiI to label TG neurons innervating the TMJ and showed that both DiI-positive neurons and Nav1.7-positive neurons were mainly located in the rostral area of the mandibular division adjacent to the maxillary division [22]; thus, the rostral area in TG was the focus for our experiments.

### 2.12. Statistical Analysis

Statistical analysis was performed using SPSS 13 for Windows. All data were presented as mean ± SD. Comparisons between two groups were analyzed using an independent samples *t*-test, and comparisons between more than two groups were analyzed using by one-way analysis of variance (ANOVA) followed by Tukey's multiple comparisons test. Any value of *P* < 0.05 was considered to indicate statistical significance.

## 3. Results

### 3.1. Attenuation of Sodium Currents in TG Neurons by P4

**Figure 3:**
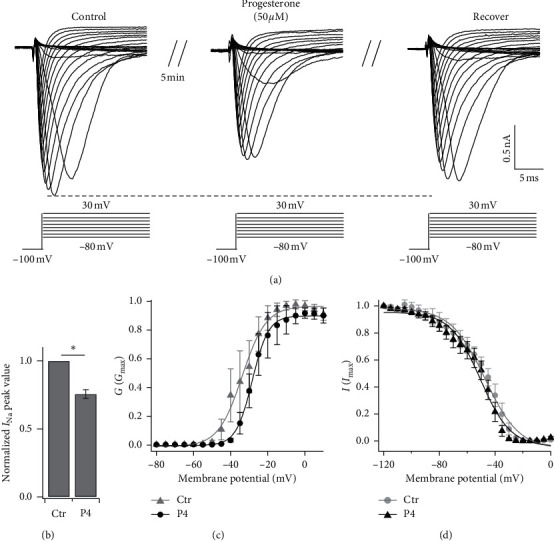
Progesterone attenuated sodium currents in freshly isolated TG neurons. (a) Representative traces showing that extracellular application of progesterone (P4, 50 *µ*M) decreases sodium currents after 5 min reversibly in freshly isolated TG neurons in response to 50 ms depolarization from −80 mV to 30 mV from an holding potential of −100 mV with an increment of 5 mV every 5 s. (b) Statistical data showing that 5 min treatment with P4 (50 *µ*M) attenuate the peak value of sodium currents (*n* = 6) in freshly isolated TG neurons. ^*∗*^*P* < 0.05 versus the control group. (c) The voltage-dependent activation curve of sodium channels in TG neurons before and after P4 application. (d) The steady-state inactivation curve of sodium channels in TG neurons before and after P4 application.

Sodium currents in TG neurons (15–25 *µ*m in diameter) were detected by membrane depolarization over a range from −80 mV to 30 mV with 5 mV steps at an interval of 5 s at 50 ms for each step after beginning from a holding potential of −100 mV. Extracellular application of P4 (50 *µ*M) reversibly attenuated sodium currents in freshly isolated TG neurons (Figure 3(a)), and it decreased the peak value by approximately 21% in 5 min (*n* = 6) (Figure 3(b)). To study the effect of P4 on steady-state activation of sodium currents, the conduction-voltage curves were fit to the Boltzmann function; P4 negatively shifted the activation curve to the right by about 5 mV (control: *V*_1/2_ = −33.78 ± 0.75 mV, *k* = 6.43 ± 0.63; P4: V_1/2_ = −28.36 ± 0.21 mV, *k* = 4.37 ± 0.19; *n* = 5, Figure 3(c)). In contrast, the channel inactivation curve fit by the Boltzmann function was little affected by P4 (control: *V*_1/2_ = −47.18 ± 1.12 mV, *k* = 12.2 ± 1.03; P4: *V*_1/2_ = −50.4 ± 1.24 mV, *k* = 10.77 ± 1.14; *n* = 3, Figure 3(d)).

### 3.2. Confirmation of Ovariectomy and Progesterone Administration

Serum levels of P4 were measured to confirm the effectiveness of ovariectomy and P4 replacement. As shown in Figure 1(a), serum levels of P4 in the ovariectomized groups increased dose dependently, as expected, with the lowest level (4.3 ± 1.5 ng/ml) in the 0 *μ*g P4 group and the highest (25.6 ± 3.2 ng/ml) in the 700 *μ*g P4 group, respectively. Serum level of P4 in the control group (16.1 ± 6.5 ng/ml) was higher than that in the 0 *μ*g P4 group (*P* < 0.05), was comparable with that in the 350 *μ*g P4 group (17.2 ± 3.0 ng/ml; *P* > 0.05), and was lower than that in the 700 *μ*g P4 group (*P* < 0.05). In addition, serum levels of P4 in all ovariectomized groups were within the physiological level observed over the estrous cycle of normal female rats [30].

### 3.3. P4 Downregulated Nav1.7 Expression in TG and Attenuated the Sensitivity of TMJ Nociception

The head withdrawal threshold, which assays for TMJ nociception, was highest in the 700 *μ*g P4 group (*P* < 0.05) (Figure 1(b)), while Nav1.7 mRNA and protein expression was lowest in the 700 *μ*g P4 group (*P* < 0.05) (Figures 1(c) and 1(d)). These results suggest that the serum level of P4 may be related to the downregulation of Nav1.7 expression and decreased sensitivity to TMJ nociception.

### 3.4. P4 Dose Dependently Attenuated Allodynia of Inflamed TMJ and TMJ Inflammation-Induced Nav1.7 Expression in TG of Ovariectomized Rats

The effectiveness of ovariectomy and P4 replacement was confirmed by the measurement of serum P4. As shown in Figure 2(a), serum levels of P4 were elevated in a dose-dependent manner. As shown in Figure 2(b), the head withdrawal threshold significantly decreased in the sham + CFA group compared with that in the control group (*P* < 0.05). However, the TMJ inflammation-induced decrease of the head withdrawal threshold in the ovariectomized groups was attenuated by P4 in a dose-dependent manner (*P* < 0.05), indicating that P4 replacement could partially reverse TMJ inflammation-induced downregulation of the head withdrawal threshold. As shown in Figures 2(c) and 2(d), Nav1.7 mRNA and protein expression were significantly upregulated in the sham + CFA group compared with the control group (*P* < 0.05), and TMJ inflammation-induced Nav1.7 expression in the ovariectomized groups was partially inhibited by P4 in a dose-dependent manner (*P* < 0.05). This result demonstrated that P4 could prevent TMJ inflammation-induced Nav1.7 expression in TG and attenuate TMJ inflammation-induced allodynia.

### 3.5. Blocking PR Partially Reversed the Repressive Effect of P4 on Allodynia from TMJ Inflammation and Nav1.7 Expression in TG

**Figure 4:**
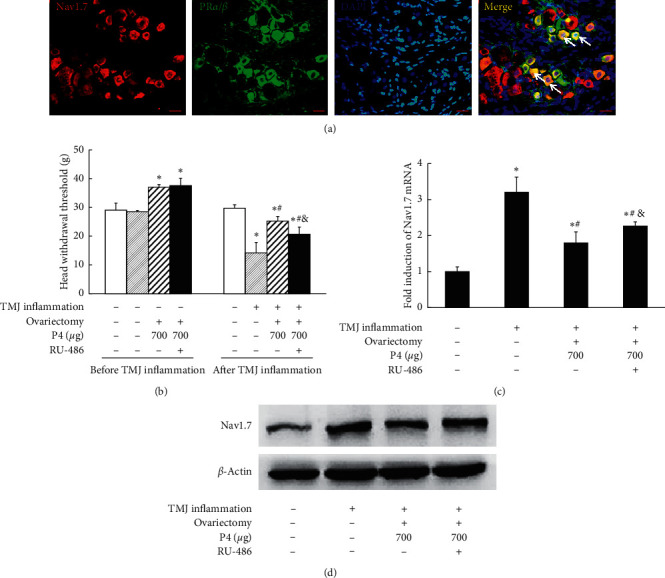
Blocking progesterone receptors partially reversed the effect of P4 on TMJ hyperalgesia and Nav1.7 expression. (a) Representative immunofluorescence of Nav1.7 and PR*α*/*β* in TG. There was colocalization between Nav1.7 and PR*α*/*β* (yellow, indicated by arrow) in TG neurons, bars = 20 *μ*m. (b) Head withdrawal threshold. RU486 partially inhibited the repressive effect of P4 on head withdrawal threshold. (c) Real-time PCR analysis for Nav1.7 expression in TG. After induction of TMJ inflammation, Nav1.7 mRNA expression was significantly increased in the sham rats, and 700 *μ*g P4 partially repressed TMJ inflammation-induced Nav1.7 mRNA expression. RU486 partially inhibited the repressive effect of P4 on Nav1.7 mRNA expression. (d) Representative immunoblotting for Nav1.7 expression in TG. Nav1.7 protein expression corresponded to the mRNA expression pattern. *β*-Actin served as an internal control for equal loading. ^*∗*^*P* < 0.05 versus the control group; ^#^*P* < 0.05 versus the sham group; ^&^*P* < 0.05 versus 700 *μ*g P4 without the RU486 group. Data are expressed as mean ± SD.

To examine whether P4 exerts its regulatory role by mediating Nav1.7 expression through effects on PR, the PR antagonist RU-486 was applied. As shown in Figure 4(a), there was colocalization between Nav1.7 and PR*α*/*β* in TG neurons. Serum levels of P4 were detected to confirm the effectiveness of P4 replacement (data not shown). As shown in Figure 4(b), the head withdrawal threshold was significantly decreased in the inflammation group compared with the control group (*P* < 0.05), and 700 *μ*g P4 partially attenuated TMJ inflammation-induced downregulation of the head withdrawal threshold. RU-486 partially attenuated the effects of P4 on head withdrawal threshold (*P* < 0.05). As shown in Figures 4(c) and 4(d), Nav1.7 mRNA and protein expression in the inflammation group were significantly upregulated compared with the control group (*P* < 0.05), while Nav1.7 mRNA and protein expression in the 700 *μ*g P4 group were significantly downregulated compared with the inflammation group. Thus, inflammation-induced Nav1.7 mRNA and protein expression were attenuated by 700 *μ*g P4. Moreover, Nav1.7 mRNA and protein expression in the 700 *μ*g P4 + RU486 group were significantly upregulated compared with the 700 *μ*g P4 group and downregulated compared with the inflammation group, indicating that RU-486 partially attenuated the repressive effect of P4 on Nav1.7 expression (*P* < 0.05).

## 4. Discussion

**Figure 5:**
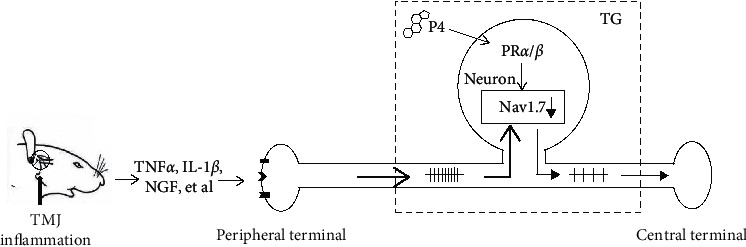
A model diagram of progesterone attenuating allodynia of inflamed temporomandibular joint (TMJ) through modulating voltage-gated sodium channel 1.7 in trigeminal ganglion. TMJ inflammation induces expressions of cytokines, such as TNF*α*, IL-1*β*, and NGF. These cytokines will act on the nerve endings, initiate impulse, and induce Nav1.7 expression in the TG. P4 may downregulate trigeminal ganglionic Nav1.7 expressions, which reduce the stimuli in the neurons, through progesterone receptors (PR*α*/*β*), and then attenuates allodynia of inflammatory TMJ.

In this study, we have shown that progesterone was able to downregulate Nav1.7 expression in TG, thereby attenuating allodynia from TMJ inflammation. We found that progesterone reversibly attenuated sodium currents in freshly isolated TG neurons. These effects were correlated with progesterone-mediated dose-dependent downregulation of Nav1.7 expression in TG and reduced sensitivity of TMJ nociception. Moreover, progesterone dose-dependently attenuated allodynia of inflamed TMJ and TMJ inflammation-induced Nav1.7 expression in TG. Finally, the progesterone receptor antagonist RU-486 partially attenuated the effects of progesterone on the mechanical pain threshold and Nav1.7 expression. We summarized the results and proposed a model that progesterone could attenuate allodynia of inflammatory TMJ through modulating trigeminal ganglionic Nav1.7 in Figure 5. TMJ inflammation induced expressions of cytokines, such as TNF*α*, IL-1*β*, and NGF [31]. These cytokines will act on the nerve endings, initiate impulse, and induce Nav1.7 expression in the TG. Progesterone may downregulate trigeminal ganglionic Nav1.7 expressions, which reduce the stimuli in the neurons, through progesterone receptors (PR*α*/*β*) and then attenuates allodynia of inflammatory TMJ. These findings add to our understanding of the complex role of sex hormones on TMJ nociception and may in part explain why women experience some amelioration of TMD-related pain during pregnancy.

Progesterone-induced changes in voltage-gated sodium currents (INa) in TG neurons may contribute to inhibiting the excitability of neurons. The input from peripheral nociceptive stimulation depends on the presence of INa, and TMJ inflammation may increase the excitability of TMJ afferents [32]. Moreover, when neuronal excitability is inhibited by blocking sodium channels with drugs such as lidocaine, pain is inhibited [33]. In this study, progesterone reversibly attenuated INa in freshly isolated TG neurons and reduced the peak values of INa. This rightward shift of activation by progesterone could lead to higher threshold depolarization, which would in turn slow activation and reduce neuronal excitability. Therefore, progesterone may play its analgesic role by depressing INa. These results are consistent with the view that progesterone decreases excitotoxicity through direct inhibition of voltage-gated sodium channels, calcium channels, and potassium channels [14, 34, 35]. Although we demonstrated in this study that the full panel of sodium channels involved in progesterone-mediated depression of INa, which sodium channels play the major role need to be further explored.

Progesterone may ameliorate TMJ inflammatory pain through Nav1.7 in TG. This finding puts forth a possible peripheral mechanism underlying the effect of progesterone on TMJ pain. In this study, we showed that progesterone could dose-dependently decrease Nav1.7 expression and the sensitivity of TMJ nociception. Nav1.7 was reported to set the gain on pain signaling [19], so it would be reasonable to believe that the downregulation of Nav1.7 expression may contribute to the attenuation of TMJ nociception and that Nav1.7 in TG might be a target for progesterone. Moreover, we showed that increasing doses of serum progesterone were associated with decreasing severity of TMJ inflammatory pain induced by CFA injection, and progesterone dose-dependently attenuated inflammation-induced trigeminal ganglionic Nav1.7 expression, indicating that progesterone may protect the inflamed TMJ from allodynia through downregulation of Nav1.7 in TG. In accordance, these results also strongly indicated a positive relationship existed between the allodynia of inflamed TMJ and Nav1.7 expression in TG. Given that the ameliorative effect of progesterone on TMJ allodynia is correlated with downregulation of Nav1.7 expression in TG, it may seem perplexing that Nav1.7 expression was not upregulated, and allodynia was not aggravated in the 0 *μ*g P4 + CFA group, in which serum progesterone was lower than the sham group (Figure 2). The reason may be that another hormone that is decreased in ovariectomized rats may play opposing role to progesterone in modulating allodynia and Nav1.7 expression. For example, estrogen has been reported to increase TMJ inflammatory pain [32, 36, 37], and our previous study showed that estradiol could aggravate allodynia of the inflamed TMJ by upregulating Nav1.7 in TG [3]. The combined effect of progesterone and estrogen, or some other hormone, on TMJ allodynia and Nav1.7 expression remains unclear.

Progesterone likely attenuated TMJ inflammation-induced allodynia and Nav1.7 expression in TG through the canonical progesterone receptor signaling pathway. Progesterone exerts its effects mainly through transcriptional regulatory mechanisms initiated by progesterone binding to PR*α* or PR*β* in the nucleus [38]. In this study, we first confirmed colocalization between Nav1.7 and PR*α* or PR*β* in TG neurons, which provided proof-of-concept that progesterone might influence Nav1.7 expression. Then, we blocked progesterone receptor with RU-486 to inhibit the repressive effect of progesterone on Nav1.7 expression and TMJ inflammatory pain. These results indeed suggest that progesterone might attenuate TMJ inflammatory pain, at least partially through progesterone receptors, and Nav1.7. RU486 antagonizes P4 action by its binding with PR allowing dimerization and binding with DNAs HREs but avoids transcription [39]. Although we pretreated with adequate RU486, the progesterone's effect was not fully reversed. There may be some other mechanism involved in progesterone-mediated downregulation of Nav1.7 expression. For example, progesterone was reported to reduce the expression of cyclooxygenase-2 (COX-2) [40], and our previous study showed Nav1.7 involvement in TMJ inflammatory pain was regulated by COX-2/PGE2 signaling [41]. Furthermore, our results showed that progesterone ameliorate TMJ allodynia, and others have shown that progesterone attenuate nociceptive behavior after excitotoxic spinal cord injury [42] and inflammation-induced thermal hyperalgesia [43]; there are some researchers who reported that cycling high level of progesterone does not reduce CFA-induced TMJ nociception [30]. These differences indicate that the roles of progesterone are still not fully understood. The complication of the roles of progesterone may be related to the complicated effects of progesterone receptors, for example, increases in the myometrial cell PR*α* to *β* ratio represses the anti-inflammatory activity of progesterone receptor-*β* [44].

In conclusion, we showed that progesterone ameliorated TMJ inflamed pain, and this analgesic effect of progesterone may act partially through the repression of TMJ inflammation-induced Nav1.7 expression in TG. These data may in part explain why women with TMD experience some amelioration of pain during pregnancy. Progesterone, by modulating Nav1.7 in TG, may represent a promising medical intervention to prevent the development of TMD pain.

## Data Availability

The data used to support the findings of this study are available from the corresponding author upon request.
